# Six‐gene Assay as a new biomarker in the blood of patients with colorectal cancer: establishment and clinical validation

**DOI:** 10.1002/1878-0261.12427

**Published:** 2019-02-18

**Authors:** Xin Shou, Yong Li, Weilei Hu, Tingting Ye, Guosheng Wang, Feng Xu, Meihua Sui, Yibing Xu

**Affiliations:** ^1^ Institute of Translational Medicine Zhejiang University School of Medicine Hangzhou China; ^2^ Department of Medical Oncology Shanghai Gongli Hospital Second Military Medical University Shanghai China; ^3^ Center for Cancer Biology and Innovative Therapeutics Key Laboratory of Tumor Molecular Diagnosis and Individualized Medicine of Zhejiang Province Clinical Research Institute Zhejiang Provincial People's Hospital People's Hospital of Hangzhou Medical College China

**Keywords:** biomarker, circulating tumor cells, colorectal cancer, RT‐PCR, Six‐gene Assay

## Abstract

Colorectal cancer (CRC) is the second most common cancer in men and the third most common cancer in women. Although long‐term survival has improved over the past 30 years, at least 50% of patients with CRC will develop metastases after diagnosis. In this study, we examined whether quantifying the mRNA of six CRC‐related genes in the blood could improve disease assessment through detection of circulating tumor cells (CTC), and thereby improve progression prediction in relapsed CRC patients. Cell spiking assay and RT‐PCR were performed with blood samples from healthy volunteers spiked with six CRC cell lines to generate an algorithm, herein called the Six‐gene Assay, based on six genes (*CEA*,* EpCAM*,* CK19*,*MUC1*,*EGFR* and *C‐Met*) for CTC detection. The CTCs of 50 relapsed CRC patients were then respectively measured by CEA Gene Assay (single‐gene assay control) and Six‐gene Assay. Subsequently, receiver operating characteristic analysis of the CTC panel performance in diagnosing CRC was conducted for both assays. Moreover, the 2‐year progression‐free survival (PFS) of all patients was collected, and the application of CEA Gene Assay and Six‐gene Assay in predicting PFS was carefully evaluated with different CTC cutoff values. Encouragingly, we successfully constructed the first multiple gene‐based algorithm, named the Six‐gene Assay, for CTC detection in CRC patients. Six‐gene Assay was more sensitive than CEA Gene Assay; for instance, in 50 CRC patients, the positive rate of Six‐gene Assay in CTC detection was 82%, whereas that of CEA Gene Assay was only 70%. Moreover, Six‐gene Assay was more sensitive and accurate than CEA Gene Assay in diagnosing CRC as well as predicting the 2‐year PFS of CRC patients. Statistical analysis demonstrated that CTC numbers measured by Six‐gene Assay were significantly associated with 2‐year PFS. This novel Six‐gene Assay improves the definition of disease status and correlates with PFS in relapsed CRC, and thus holds promise for future clinical applications.

**Abbreviations**
CRC: colorectal cancer
CTC: circulating tumor cells
Ct: cycle threshold
GM: geometric mean
PBMC: peripheral blood mononuclear cells
PFS: progression‐free survival
RIN: RNA integrity numbers
ROC: receiver operating characteristic
TRC: transcription–reverse transcription concerted reaction

## 1 Introduction

Colorectal cancer (CRC) is the second most common cancer in men and the third most common cancer in women (Siegel *et al*., 2018). The incidence rates are higher in developed countries than in developing ones. Although its long‐term survival has been improved over the past 30 years with multi‐modality therapy, at least 50% of patients with CRC will develop metastases after diagnosis, leading to a poor prognosis with a 5‐year overall survival rate of 12.5% (Siegel *et al*., 2017; Van Cutsem *et al*., 2014). Therefore, new therapeutic strategies are needed to improve the outcome of patients with relapsed CRC. However, these likely will be based on studies that include sequencing of CRC cells to identify actionable targets, defining targets in the tumor microenvironment attributed to tumor growth and resistance to therapy, and evaluating disease burden accurately to predict response and prognosis (Grasso *et al*., 2018; Punt *et al*., 2017; Sanchez‐Lopez *et al*., 2016; Stadler *et al*., 2016; Yaeger *et al*., 2018).

It has been indicated that evaluation of disease burden by quantifying circulating tumor cells (CTC) in blood may contribute to response assessment and prognostication (Haber and Velculescu, 2014; Lalmahomed *et al*., 2015). For instance, studies have been performed at the time of diagnosis or during initial therapy prior to disease progression using CellSearch® (EpCAM antigen‐based) and Transcription‐Reverse Transcription Concerted Reaction (TRC, CEA mRNA‐based) methods. These methods have provided useful information on CRC patients (Gorges *et al*., 2016; Sato *et al*., 2012). However, due to the heterogeneity of CTC, single antigens alone, such as EpCAM or CEA, are not sufficient to capture all the CTC in blood. This could explain why CellSearch® and TRC methods generate much lower yields of CTC than expected. As reported previously, only 30–40% of CRC patients harbor three or more CTC per 7.5 mL of blood using the immunomagnetic CellSearch® detection system (Gorges *et al*., 2016), and only 60% of CRC patients were shown to be positive in the CTC test with blood samples using the TRC method (Sato *et al*., 2012). Therefore, a combined analysis of more than one gene could improve the sensitivity of CTC detection by increasing the range of CTC markers, giving an important advantage in view of the well‐known phenotypic heterogeneity of CTC.

Dozens of CRC biomarkers have been identified by different technologies, e.g. microarray profiling (Yanagawa *et al*., 2001), high‐throughput gene sequencing (Kim *et al*., 2011) and mutation detection (Mao *et al*., 2015). As a result, several multiple gene‐based assays have been reported for diagnosis of primary CRC tumors, as well as for the prognostic prediction of CRC (Ning *et al*., 2015; Okugawa *et al*., 2015; Shimada *et al*., 2012). Despite the fact that CTC fall off primary tumors, they carry gene expression signatures different from those of the primary tumors (Chaffer and Weinberg, 2011). This suggests that biomarkers from primary tumors may not satisfy the demand to quantify CTC because of their heterogeneity in gene expression. In this study, after a careful evaluation of the expression level of CRC‐related biomarkers in patient tissues and CRC cell lines (de Albuquerque *et al*., 2012; Cayrefourcq *et al*., 2015; Cohen *et al*., 2006; Gasch *et al*., 2013; Iinuma *et al*., 2011), we selected six genes—*CEA*,* EpCAM*,*CK19*,*MUC1*,*EGFR* and *C‐Met*—and evaluated their potential as biomarkers for CTC detection and prognostic prediction of CRC. We successfully constructed the first multiple gene‐based algorithm, here denoted the Six‐gene Assay, for quantifying the number of CTC in CRC patients based on mRNA level of the above six genes. Moreover, using CEA Gene Assay as a single‐gene assay control, we demonstrated that this novel Six‐gene Assay is superior in both CTC detection and prediction of progression‐free survival (PFS) in CRC patients.

## 2 Material and methods

### 2.1 Cell cultures

Human colon cancer cell lines HCA‐7, LoVo, SW620, RKO, SW1116 and SW48 were purchased from the American Type Culture Collection (ATCC, Manassas, VA, USA) and cultured according to the recommended protocols. In brief, SW48, SW620 and SW1116 cell lines were cultured in flasks containing RPMI‐1640 medium, LoVo cell line was cultured in Ham's F‐12K medium, HAC‐7 cell line was cultured in Dulbecco's modified Eagle's medium, and RKO cell line was cultured in Eagle's minimal essential medium. All culture media were supplemented with 10% FBS (Gibco, Grand Island, NY, USA). These CRC cell lines were selected as they express different levels of six validated genes (*CEA*,* EpCAM*,*CK19*,*MUC1*,*EGFR* and *C‐Met*).

### 2.2 Patients and healthy volunteers

The study was designed in accordance with the Declaration of Helsinki, and was performed following the protocols and informed consent documents approved by the Institutional Review Board of Shanghai Gongli Hospital (Shanghai, China). Fifty relapsed patients with stage III or stage IV CRC at diagnosis were included between January 2015 and January 2017 (Table 1). All patients were treated with chemotherapy and none of them underwent radical surgery for the tumors after relapse. Patients were monitored by CT scanning for tumor progression. In addition, 10 healthy volunteers who were clear of polyps under colonoscopy and had no family history of CRC were enrolled as controls. Written consent forms were obtained from all patients and healthy volunteers prior to study enrollment.

**Table 1:** Clinicopathologic characteristics of patients with CRC

Variable	Category	No. of patients/age in years
Sex	Male	28
	Female	22
Age at diagnosis	Median in years	67 (45–78)
Stage at diagnosis	Stage III	38
	Stage IV	12
Metastasis	To liver	12
	To other organs	18
	Unknown/not done	20
Chemotherapy	Yes	50
	No	0
Surgery after relapse	Yes	0
	No	50

### 2.3 Sample processing and RNA extraction

A peripheral blood sample (5.0 mL) was drawn from each patient or healthy volunteer into PAXgene vials (BD, Biosciences, San Jose, CA, USA). All the samples were maintained at room temperature (20–25 °C) and processed within 72 h after collection. Mononuclear cells from each peripheral blood sample were isolated by density separation with Ficoll‐Paque (GE Healthcare, Little Chalfont, Buckinghamshire, UK). RNA was extracted from mononuclear cells using an RNeasy® Plus Micro Kit (Qiagen, Duesseldorf, Germany) according to the manufacturer's instructions. The extracted RNA was eluted with 20 μL of RNase‐free water. For RNA quality control, the ratios of the absorbance (*A*) at wavelengths of 260 and 280 nm were determined for all samples, which were between 1.90 and 2.10. To further determine the quality of RNA, an RNA integrity test was performed as previously described (Schroeder *et al*., 2006). The average RNA integrity number (RIN) of the specimens used in this study was 9.1 (range 8.0–10.0).

### 2.4 Reverse transcription polymerase chain reaction (RT‐PCR)

Total RNA extracted from peripheral blood mononuclear cells (PBMC) was reverse transcribed using M‐MLV reverse transcriptase (Promega, Madison, WI, USA) according to the manufacturer's instructions. RT‐PCR assays were then performed to assess mRNA expression level of *CEA*,* EpCAM*,*CK19*,*MUC1*,*EGFR* and *C‐Met* in 20 μL reaction mixture using SYBR Green PCR Master Mix (Takara, Tokyo, Japan) and the ViiA™ 7 Real‐Time PCR System (Applied Biosystems, Foster City, CA, USA). *B2M* and *GAPDH* were used as internal control genes. The specific primers of 5′‐GCAGCTGTCCAATGACAACA‐3′ and 5′‐GGACGGTAATAGGTGTATGA‐3′ were used to amplify the human CEA coding region; the specific primers of 5′‐TGCTGGAATTGTTGTGCTGG‐3′ and 5′‐AAGATGTCTTCGTCCCACGC‐3′ were used to amplify the human EpCAM coding region; the specific primers of 5′‐GGTGAAGATCCGCGACTGGT‐3′ and 5′‐CGTCTCAAACTTGGTTCGGA‐3′ were used to amplify the human CK19 coding region; the specific primers of 5′‐CTCTCCAATATTAAGTTCAGG‐3′ and 5′‐GAAAGGAAATGGCACATCACT‐3′ were used to amplify the human MUC1 coding region; the specific primers of 5′‐TGTGCCCACTACATTGACGG‐3′ and 5′‐TAGGCCCATTCGTTGGACAG‐3′ were used to amplify the human EGFR coding region; and the specific primers of 5′‐TTGGAAATGAGAGCTGCACCT‐3′ and 5′‐TCGGCGAAATACTTGTTATT‐3′ were used to amplify the human C‐Met coding region. The specific primers of 5′‐TGTCTTTCAGCAAGGACTGGT‐3′ and 5′‐TCATCCAATCCAAATGCGGC‐3′ were used to amplify the human B2M coding region, and the specific primers of 5′‐GGAGCCAAAAGGGTCATCATCT‐3′ and 5′‐GAGCGGAATCCACCTCCACACT‐3′ were used to amplify the human GAPDH coding region.

### 2.5 Cell spiking assay and algorithm development

After using trypsin to remove the cell adhesion ability, the number of cancer cells was counted four times and the means determined respectively for all six CRC cell lines mentioned above. Predetermined numbers (1, 10, 100 and 1000, respectively) of cells from each CRC cell line were spiked into 1.0 mL of peripheral blood from each healthy volunteer. The blood samples were then further processed by Ficoll‐Paque gradient separation, RNA extraction and RT‐PCR as described above. Data obtained from spiking assay and RT‐PCR were used to construct an algorithm based on the mRNA expression of the above six genes in order to quantify the number of CTC in blood samples. Specifically, we determined the geometric mean (GM) value of cycle thresholds (Ct) for six genes involved in the algorithm.

### 2.6 CEA Gene Assay and Six‐gene Assay for CTC detection

CEA Gene Assay and Six‐gene Assay based on mRNA expression of corresponding genes were performed by RT‐PCR with samples prepared from spiked cell cultures and PBMC as described previously (Sato *et al*., 2012). Briefly, the six CRC‐associated genes (*CEA*,* EpCAM*,* CK19*,* MUC1*,* EGFR* and *C‐Met*) and housekeeping genes *B2M* and *GAPDH* were quantified by RT‐PCR with optimized primer sets. The Ct value for each gene was the cycle number where the amplification signal reached a threshold of 0.4 over baseline, and Ct of 40 was assigned when this threshold was not reached by the 40th cycle. Together with the Ct value from CEA Gene Assay and Six‐gene Assay, algorithms were used to quantify the number of CTC.

### 2.7 CEA Gene Assay and Six‐gene Assay for prediction of 2‐year PFS

To evaluate and compare the sensitivity and accuracy of CEA Gene Assay and Six‐gene Assay in predicting PFS, the 2‐year PFS of all 50 CRC patients were collected. With the number of CTC of each CRC patient respectively calculated by CEA Gene Assay and Six‐gene Assay, patients were divided into two groups having CTC levels less than or equal to the selected cutoff value, and those having CTC levels greater than the selected cutoff value. Time‐dependent covariate Cox regression was used to analyze the relation between the amount of CTC and PFS.

### 2.8 Statistical analysis

Statistical analysis of the obtained data was carried out using the spss software package, release 12.0.1 (IBM, Armonk, NY, USA). Raw data were entered into excel (Microsoft, Redmond, WA, USA) files and converted automatically into the statistical packages. Spearman's rank correlation coefficient was introduced to assess the association between CEA Gene Assay and Six‐gene Assay. CTC panel performance of CEA Gene Assay and Six‐gene Assay in diagnosing CRC was calculated at different CTC number variances and functioned as a surrogate marker for constructing receiver operating characteristic (ROC) curves, where area under the ROC curve (AUC) reflects diagnostic capability. Time‐dependent covariate Cox regression was used to analyze the relation between the amount of CTC and PFS. *P* values were based on the likelihood ratio test, and *P *<* *0.05 was considered statistically significant.

## 3 Results

### 3.1 Characteristics of patients

All 50 patients enrolled in this study had a histologically confirmed diagnosis of CRC (Table 1). The patient cohort included 28 male (56%) and 22 female (44%) patients, with an average age of 67 years at diagnosis (range 45–78 years). Thirty‐eight patients (76%) were stage III and 12 patients (24%) were stage IV CRC. Thirty patients (60%) had metastases: 12 of these patients (24%) had tumors located in the liver and 18 patients (36%) tumors located in other organs, not liver.

### 3.2 Establishment of CTC algorithms for CEA Gene Assay and Six‐gene Assay

Through cell spiking assay using different but known amounts of SW1116, SW48, HCA‐7, LoVo, SW620 and RKO cancer cells, and subsequent RT‐PCR, we successfully established the following CTC equations for CEA Gene Assay and Six‐gene Assay. Based on previous studies (Marachelian *et al*., 2017; Vandesompele *et al*., 2002), a summary ΔCt for the six tested genes was calculated by subtracting the GM of the Ct for two reference genes from the GM of the Ct for the six genes. With a direct correlation between CTC numbers and ΔCt (Six‐gene) value, this CTC quantitative equation and corresponding transformation table (Table 2) allow us to calculate CTC numbers by the ΔCt (Six‐gene) value of unknown blood samples. For CEA Gene Assay, ΔCt (CEA) was calculated in a similar way, with only CEA gene included in the equation and transformation table (Table 3).

**Table 2:** Six‐gene Assay transformation table

ΔCt (Six‐gene)	CTC number (/mL)
≥ 16.0	< 1
14.8–15.9	1
13.6–14.7	2
12.4–13.5	3
11.2–12.3	4
10.0–11.1	5
8.8–9.9	6
7.6–8.7	7
6.4–7.5	8
5.2–6.3	9
4.0–5.1	10
< 4	> 10

**Table 3:** CEA Gene Assay transformation table

ΔCt(CEA)	CTC number (/mL)
≥ 16.0	< 1
14.8–15.9	1
13.5–14.7	2
12.2–13.4	3
10.9–12.1	4
9.6–10.8	5
8.3–9.5	6
7.0–8.2	7
5.7–6.9	8
4.4–5.6	9
3.0–4.3	10
< 3	> 10

(I) CTC equation for Six‐gene Assay:

$$\begin{array}{cc} \Delta \text{Ct(Six‐gene)}= & {}^{6}\sqrt{\text{Ct(CEA)}\times \text{Ct(EpCAM)}\times \text{Ct(CK19)}\times \text{Ct(MUC1)}\times \text{Ct(EGFR)}\times \text{Ct(C‐met)}}-\\ & {}^{2}\sqrt{\text{Ct(B2M)}\times \text{Ct}(\beta -\text{actin})} \end{array}$$

(II) CTC equation for CEA Gene Assay:

$$\Delta \mathrm{Ct}(\mathrm{CEA})=\mathrm{Ct}(\mathrm{CEA})-{}^{2}\sqrt{\mathrm{Ct}(B2M)\times \mathrm{Ct}(\beta -\mathrm{actin})}$$

### 3.3 Superiority of Six‐gene Assay over CEA Gene Assay in CTC detection of CRC patients

**Figure 1:**
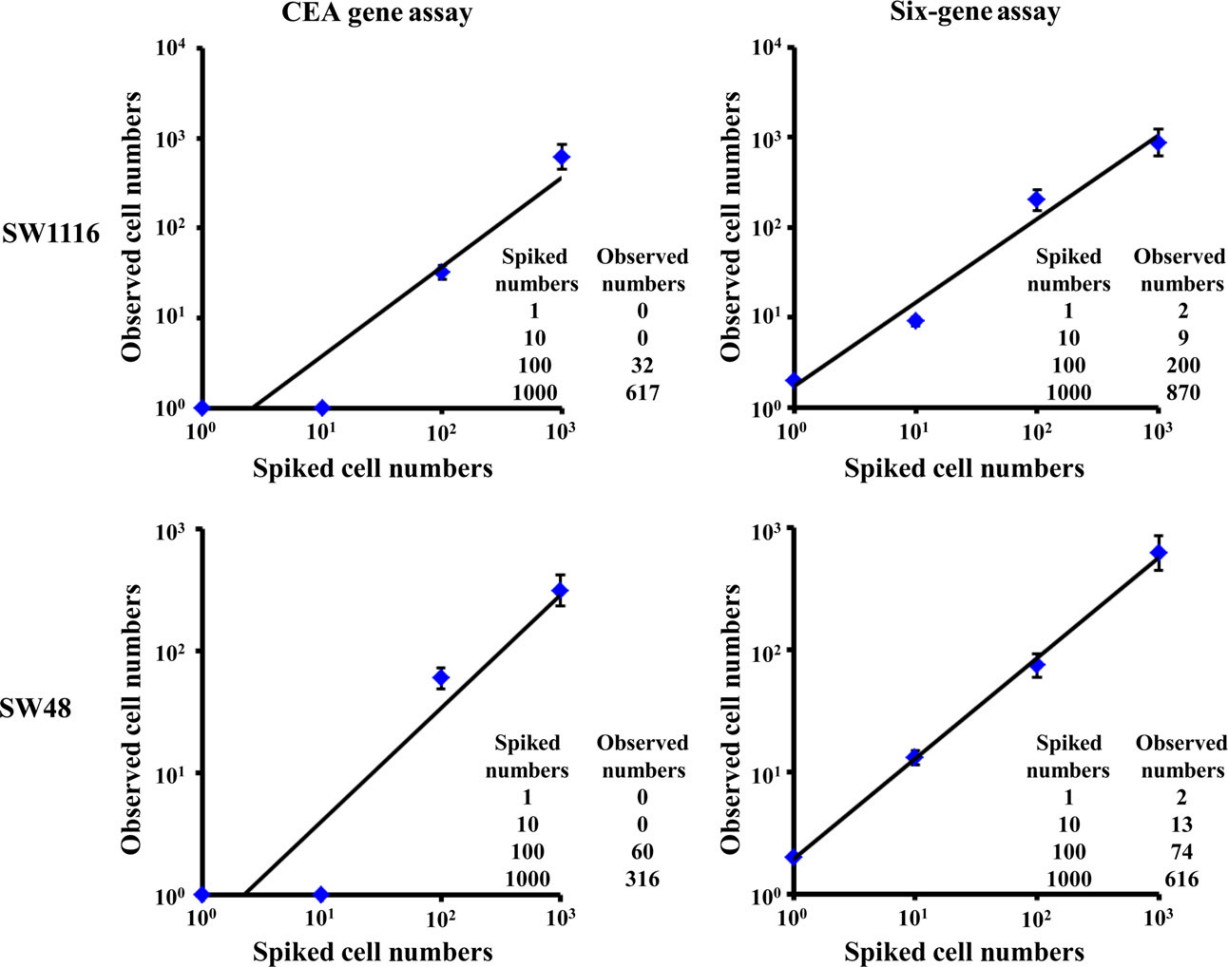
Evaluation of cell detection efficiency of CEA Gene Assay and Six‐gene Assay. A dilution series of cells (1, 10, 100 and 1000) from CRC cell lines SW1116 and SW48 were respectively spiked in 1.0 mL of peripheral blood from a healthy donor. Blood samples were further processed by Ficoll‐Paque gradient separation, RNA extraction and real‐time quantitative PCR. The plot represents number of cells spiked versus number of cells observed. The recovery of spiked numbers of CRC cells was measured by CEA Gene Assay and Six‐gene Assay based on the mRNA expression of corresponding genes in CRC cell lines. Each error bar represents mean ± SD. Inset tables provide detailed numbers for each dilution.

After establishment of the above CEA Gene Assay and Six‐gene Assay algorithms, we applied them to evaluate CTC in 10 healthy donors. Our data demonstrated that these samples were all negative for CTC with both CEA Gene Assay and Six‐gene Assay. Next, six CRC cell lines were spiked respectively into blood samples of 10 healthy donors, followed by RT‐PCR and subsequent CEA Gene Assay and Six‐gene Assay, respectively. Six‐gene Assay showed a significantly higher sensitivity than CEA Gene Assay; for instance, Six‐gene Assay could detect one CRC cell among 10^6^ PBMC in all the tested cell lines, but although CEA Gene Assay could detect one CRC cell among 10^6^ PBMC in HCA‐7, LoVo, SW620 and RKO cell lines, it could not detect CRC cells < 100 among 10^6^ PBMC in SW1116 and SW48 cell lines (Figs 1 and S1). On the other hand, compared with CEA Gene Assay, the observed CRC cell numbers were closer to the spiked numbers in the dilution series (1, 10, 100 and 1000 cells/mL of blood) in Six‐gene Assay.

**Figure 2:**
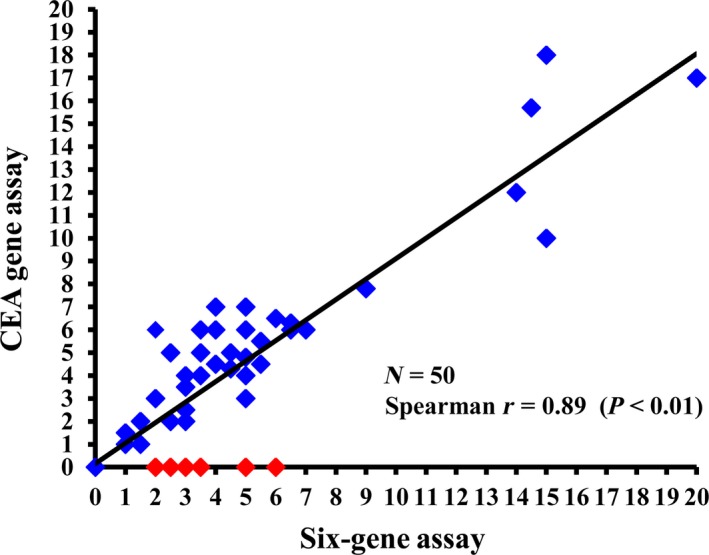
Correlation between CEA Gene Assay and Six‐gene Assay in CTC detection. CEA Gene Assay and Six‐gene Assay were performed with blood specimens from 50 relapsed CRC patients. The CTC numbers respectively measured by Six‐gene Assay (*x*‐axis) and CEA Gene Assay (*y*‐axis) for each patient are shown on the chart. Patients with CTC detected by Six‐gene Assay but undetectable by the CEA Gene Assay are indicated with red dots. Spearman's *r* = 0.89, *P *<* *0.01.

To confirm these *in vitro* findings, we further determined the superiority of Six‐gene Assay to CEA Gene Assay with clinical samples in which patient samples were defined as CTC‐positive once one or more cancer cells were detected. Forty‐one samples (82%) were CTC‐positive with Six‐gene Assay (CTC numbers: 5.2 ± 4.4) (Fig. 2). Although there was a significant correlation between Six‐gene Assay and CEA Gene Assay (Spearman *r* = 0.89, *P *<* *0.01), only 35 samples (70%) were found to be CTC‐positive in CEA Gene Assay, six patients being false‐negative in this single gene‐based CTC test (highlighted as red points in Fig. 2).

### 3.4 Diagnostic performance of CEA Gene Assay and Six‐gene Assay

**Figure 3:**
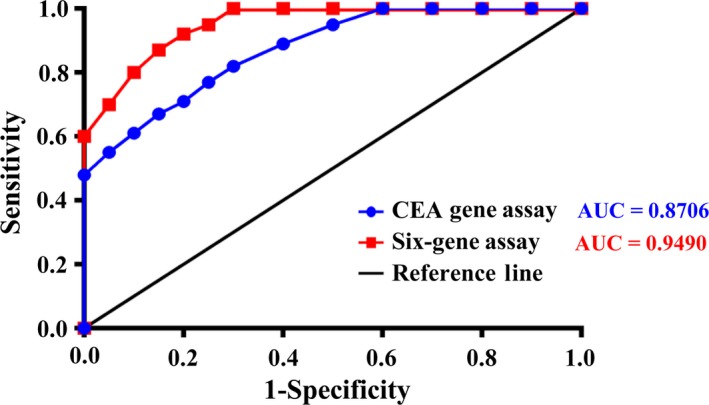
ROC analysis of the CTC panel performance in diagnosing CRC was conducted for CEA Gene Assay and Six‐gene Assay. CEA Gene Assay and Six‐gene Assay were performed with blood specimens from 50 relapsed CRC patients. The specificity (*x*‐axis) and sensitivity (*y*‐axis) of CEA Gene Assay (blue) and Six‐gene Assay (red) were shown on the chart, respectively.

To evaluate further the value of CEA Gene Assay and Six‐gene Assay in diagnosing CRC, ROC curves were constructed to determine the differences between these two assays (Fig. 3). The AUC of ROC curve of CEA Gene Assay was 0.8706, whereas that of Six‐gene Assay was 0.9490. According to the ROC curve, a relative level of 5 CTC was defined as the optimal cutoff value in CEA Gene Assay for distinguishing CRC patients from healthy donors. At this cutoff value for CTC detection, the sensitivity and specificity of CEA Gene Assay were 77% and 85%, respectively. However, the relative number of CTC defined as the optimal cutoff value for Six‐gene Assay was 4. At this cutoff value, the sensitivity and specificity of Six‐gene Assay were 87% and 85%, respectively. These data further indicated the significantly higher sensitivity of Six‐gene Assay than CEA Gene Assay.

### 3.5 Superiority of Six‐gene Assay to CEA Gene Assay in predicting 2‐year PFS of CRC patients

**Figure 4:**
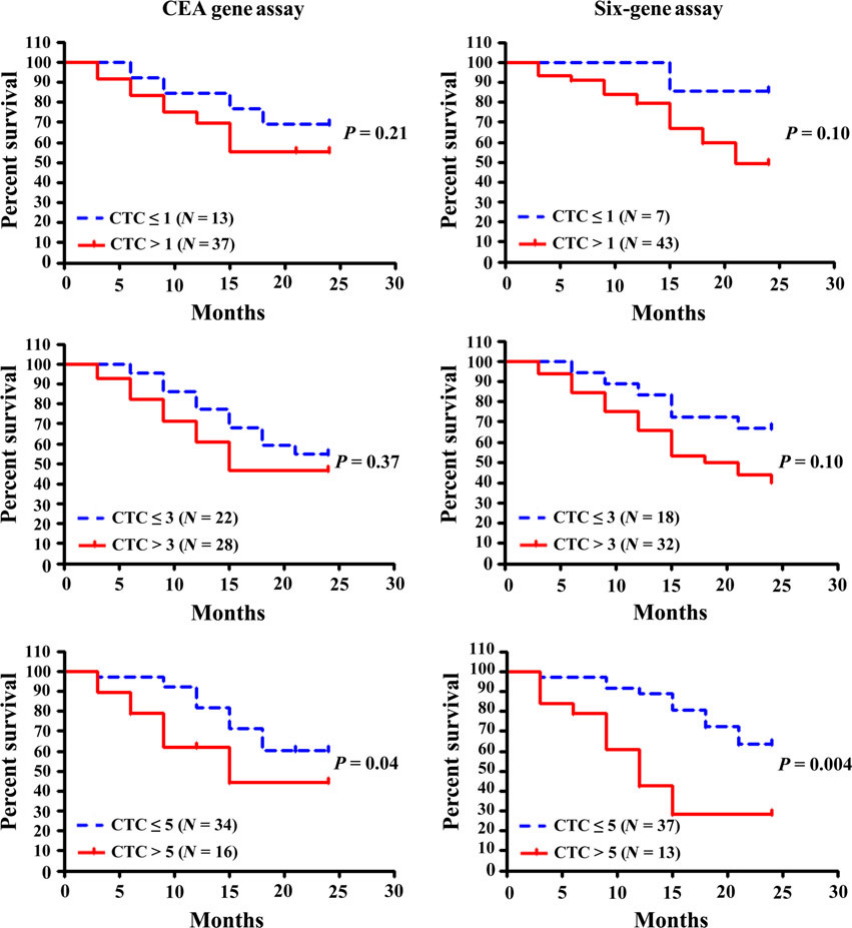
Survival analysis plots for 2‐year PFS of 50 CRC patients. Time‐dependent covariate Cox regression was used to analyze the relation between the amount of CTC and 2‐year PFS. The patients were categorized according to three different cutoff values (CTC = 1, 3 and 5, respectively), the cutoff value of 5 being approximately the median of detectable CTC values in Six‐gene Assay. *P* values in the figures correspond to the categorical univariate likelihood ratio test. *N* = number of unique patients.

To determine their roles in predicting prognosis of CRC patients, univariate analysis of CTC and 2‐year PFS was performed for both CEA Gene Assay and Six‐gene Assay (Fig. 4). CTC detection results were classified with three different cutoff values (CTC = 1, 3 and 5, respectively), where the cutoff value of 5 was approximately the median of detectable CTC values in Six‐gene Assay. As indicated in Fig. 3, the cutoff value of 5 resulted in wider separations between groups (CTC > 5, CTC ≤ 5) compared with those at cutoff values of 1 and 3 in both CEA Gene Assay and Six‐gene Assay. Consistent with this observation, the difference of PFS between CTC ≤ 5 and CTC > 5 was statistically significant in both assays (*P *=* *0.04 and 0.004, respectively), whereas those at cutoff values of 1 and 3 were not. These data indicate that both CEA Gene Assay and Six‐gene Assay at a CTC cutoff value of 5 possess predictive roles for disease progression and patient survival. One thing we would like to point out here is that at all three cutoff values, Six‐gene Assay led to wider separations than CEA Gene Assay, and the statistical *P* values obtained with Six‐gene Assay were 2–10 times lower than those obtained in CEA Gene Assay, both suggesting a higher sensitivity of Six‐gene Assay in predicting PFS in CRC patients.

## 4 Discussion

In clinics, several strategies, such as tumor‐specific markers, fine‐needle aspiration biopsy and diagnostic imaging systems, are used to evaluate the status of cancers and the anti‐cancer therapeutic effects. However, many of these methods are invasive and may even cause tumor metastasis (Miller *et al*., 2016). Meanwhile, micrometastasis and small metastatic lesions are usually undetectable by clinical imaging procedures (e.g. CT and MRI scans) (Bardelli and Pantel, 2017). For instance, the major limitation of CT is its low inherent contrast resolution, which is about 1 cm; thus tumors with diameters smaller than this can not be detected easily. More sensitive and less invasive tools are needed to ensure more accurate and timely diagnosis, as well as more effective treatments (Li *et al*., 2015; Surinova *et al*., 2015). In the past decade, studies on CTC have attracted great interest, and CTC value is now considered a useful marker for evaluating the tumor status, therapeutic response and prognosis of patients with multiple types of carcinoma including CRC (van Dalum *et al*., 2015; Hardingham *et al*., 2015; Lalmahomed *et al*., 2015). For instance, Cell Search® and the TRC method have been used to measure CTC in blood samples from CRC patients (Gorges *et al*., 2016; Sato *et al*., 2012). Although these two methods are reproducible, they are both single gene‐based (EpCAM‐ and CEA‐based, respectively). As a result, EpCAM‐negative CTC would be missed by Cell Search®, and CEA‐negative CTC are not detectable with the TRC method. Therefore, both strategies are not comprehensive enough, due to the complexity and heterogeneity of CTC.

To overcome the limitations of single gene‐based CTC detection, for the first time, we herein adopted six CRC‐related genes—*CEA*,* EpCAM*,*CK19*,*MUC1*,*EGFR* and *C‐Met*— to quantify CTC in CRC patients. We successfully constructed an algorithm for Six‐gene Assay using a spiking assay and RT‐PCR analysis with six CRC cell lines expressing different levels of the above six genes. *CEA* is highly expressed in CRC but it has a low expression level in normal tissues. Moreover, monitoring of serum *CEA* during chemotherapy may provide a predictor for patient survival in CRC (Allen‐Mersh *et al*., 1987). Therefore, CEA Gene Assay was used as a single gene‐based control to evaluate this novel Six‐gene Assay. The algorithms developed in this study for CEA Gene Assay and Six‐gene Assay allowed us to calculate the number of CTC in blood samples through the mRNA quantity of either *CEA* gene or the six CRC‐related genes. This is the first report on the establishment of a multiple gene‐based algorithm for CTC detection in CRC.

Furthermore, by using blood samples spiked with six CRC cell lines as well as blood samples from 50 relapsed CRC patients, we demonstrated that CTC detection using Six‐gene Assay was more accurate and sensitive than CEA Gene Assay. The Six‐gene Assay could detect as little as one cancer cell among 10^6^ PBMC for all six CRC cell lines, whereas the detection threshold for CEA Gene Assay was 100 cancer cells for SW1116 and SW48 cell lines. The cancer cell numbers determined by Six‐gene Assay were closer to the spiked numbers than was CEA Gene Assay. The superiority of Six‐gene Assay in CTC detection was further confirmed by clinical studies, as blood samples from 50 CRC patients were all positive with Six‐gene Assay but six of them were negative in CEA Gene Assay (false‐negative rate of 12% in CEA Gene Assay). We also found that Six‐gene Assay CTC panel shows better AUC compared with CEA Gene Assay, indicating better performance of Six‐gene Assay than CEA Gene Assay in diagnosing CRC. In terms of predicting prognosis of CRC, our data showed that although the difference in PFS between CTC ≤ 5 and CTC > 5 was statistically significant in both assays, Six‐gene Assay led to wider separations and much lower *P* values compared with CEA Gene Assay at all three cutoff values (CTC = 1, 3 and 5, respectively). These findings demonstrated that Six‐gene Assay is a novel, independent and more effective predictor of PFS in CRC compared with CEA Gene Assay.

In summary, Six‐gene Assay as validated in this study holds great promise as a novel and sensitive biomarker for early diagnosis, evaluation of therapeutic responses, as well as prognostic prediction in CRC patients. Compared with single gene assays, e.g. Cell Search®, the TRC method and CEA Gene Assay used in this study, Six‐gene Assay could potentially overcome the heterogeneity of CRC samples. Particularly, as the first multiple gene‐based algorithm for measuring CTC in CRC, Six‐gene Assay may provide a useful strategy or model system for future development of precision CTC detection and CTC‐related clinical practices. However, a number of factors, including chemotherapy and other types of treatment previously received by patients, may cause additional heterogeneity of CTC in patients. Therefore, some blood specimens from CRC patients may not have detectable mRNA of either a specific gene or the six genes validated in the present study, even though disease is present by standard evaluations. Integration of Six‐gene Assay with other traditional methods is necessary for precision and personalized medical treatment in CRC. Furthermore, the CRC‐relevant genes selected for analysis and the possible difference in the weight of each gene in the algorithm may need to be further optimized to cover as much CTC as possible and to improve the promising Six‐gene Assay; this could be achieved by future clinical studies including larger numbers of CRC patients.

## 5 Conclusions

This study investigated whether quantifying the expression of six CRC‐related genes in the blood could improve disease assessment through detection of CTC and thereby improve progression prediction in relapsed CRC patients. Through cell spiking assay and RT‐PCR, we successfully generated a novel algorithm, named Six‐gene Assay, based on the mRNA expression of *CEA*,* EpCAM*,*CK19*,*MUC1*,*EGFR* and *C‐Met* in six CRC cell lines. Furthermore, using CEA Gene assay as the single‐gene assay control, clinical validation of Six‐gene Assay with 50 blood samples from relapsed CRC patients demonstrated its superiority in defining disease status and predicting PFS. The Six‐gene Assay is the first multiple gene‐based algorithm for CTC detection in CRC, and thus provides a useful strategy or valuable model for CTC‐related research and clinical practices.

## Author contributions

XS, YL, MHS and YBX designed the study; XS, WLH, TTY and GSW performed the laboratory experiments; YL and FX recruited patients in the clinical study for this analysis; XS, YL, WLH, TTY, GSW and FX collected the clinical data; XS, YL, WLH, TTY, GSW and FX analyzed the data and compiled statistics; XS, MHS and YBX wrote the manuscript. All authors approved the final version of the manuscript, including the authorship list.

## Conflicts of interest

The authors declare no conflict of interest.

## Supporting information


**Fig. S1.** Evaluation of cell detection efficiency of CEA Gene Assay and Six‐gene Assay.
