# Supplementary figures and images for: Six‐gene Assay as a new biomarker in the blood of patients with colorectal cancer: establishment and clinical validation
Source: Mol Oncol. 2019 Feb 18;13(4):781–91. doi: 10.1002/1878-0261.12427 (PMC6441906; doi:10.1002/1878-0261.12427)

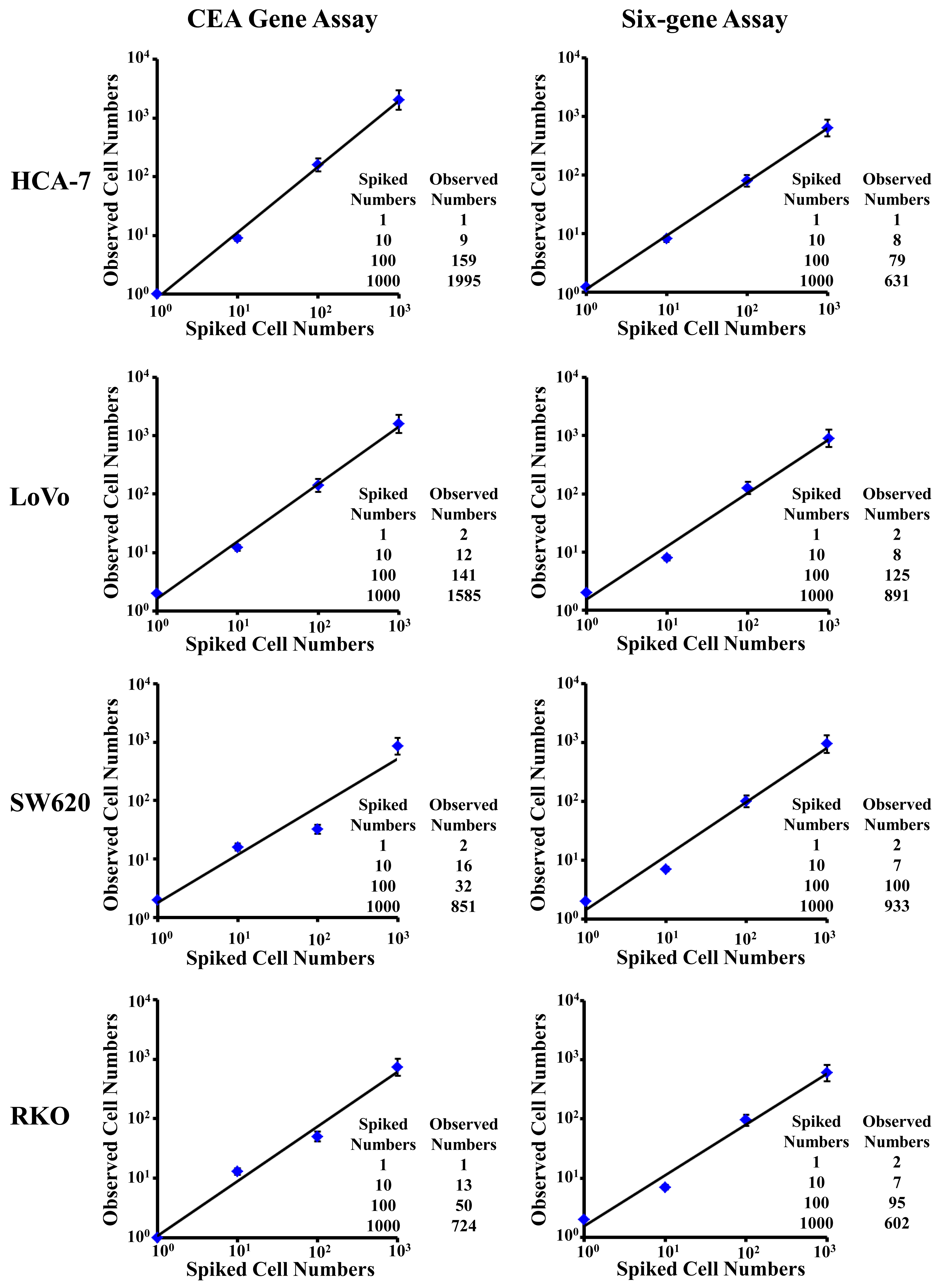

Supplement: Supplementary file 1 — Fig. S1. Evaluation of cell detection efficiency of CEA Gene Assay and Six‐gene Assay. [file MOL2-13-781-s001.tif]
